# Retraction: Hemorrhagic Infarct as a Rare Presentation of Vascular Ehlers-Danlos Syndrome in a 40-Year-Old Female: A Case Report

**DOI:** 10.7759/cureus.r177

**Published:** 2025-04-01

**Authors:** Wahab Moustafa, Taranpreet Singh, Arzoo Siddiqi, Anoushka A Sehgal, Bismil Afghan

**Affiliations:** 1 Neurosurgery, SRH Wald-Klinikum Gera, Gera, DEU; 2 Internal Medicine, Mahatma Gandhi Mission (MGM) Medical College and Hospital, Navi Mumbai, IND; 3 Internal Medicine, Dow University of Health Sciences, Karachi, PAK; 4 Internal Medicine, Mahatma Gandhi Medical College and Research Institute, Pondicherry, IND; 5 Internal Medicine, Nishtar Medical University, Multan, PAK

The Editors-in-Chief have retracted this article. An investigation by the journal found evidence of authorship manipulation. The Editors-in-Chief therefore no longer have confidence in the provenance and reliability of the article contents.

